# How does the functional diversity of frugivorous birds shape the spatial pattern of seed dispersal? A case study in a relict plant species

**DOI:** 10.1098/rstb.2015.0280

**Published:** 2016-05-19

**Authors:** Jessica E. Lavabre, Luis J. Gilarranz, Miguel A. Fortuna, Jordi Bascompte

**Affiliations:** 1 Integrative Ecology Group, Estación Biológica de Doñana, (EBD-CSIC), C/Américo Vespucio s/n, Sevilla 41092, Spain; 2 Department of Evolutionary Biology and Environmental Studies, University of Zurich, Winterthurerstrasse 190, Zurich 8057, Switzerland

**Keywords:** *Taxus baccata*, ecosystem services, gene flow, spatial network, heterogeneous landscape, modularity

## Abstract

Genetic markers used in combination with network analysis can characterize the fine spatial pattern of seed dispersal and assess the differential contribution of dispersers. As a case study, we focus on the seed dispersal service provided by a small guild of frugivorous birds to the common yew, *Taxus baccata* L., in southern Spain. We build the spatial networks of seed dispersal events between trees and seed-plots within the studied population—local network—and the spatial network that includes all dispersal events—regional network. Such networks are structured in well-defined modules, i.e. groups of tightly connected mother trees and seed-plots. Neither geographical distance, nor microhabitat type explained this modular structure, but when long-distance dispersal events are incorporated in the network it shows a relative increase in overall modularity. Independent field observations suggested the co-occurrence of two complementary groups, short- and long-distance dispersers, mostly contributing to the local and regional seed rain, respectively. The main long-distance disperser at our site, *Turdus viscivorus*, preferentially visits the most productive trees, thus shaping the seed rain at the landscape scale and affecting the local modular organization. We end by discussing how DNA barcoding could serve to better quantify the role of functional diversity.

## 1. Introduction

The relationship between biodiversity and ecosystem functioning has received a growing interest since the late 1990s [1–4]. However, experiments addressing this relationship have mostly been carried under controlled conditions [5,6] and, while these experiments have provided important insight, there is an urgent need to extend our knowledge to natural settings [2,7]. Moreover, the focus has largely been on the relationship between species richness and primary production in temperate grasslands ([8] and references therein), and our knowledge of other types of relationships or different landscapes remains limited ([2,7] but see [9]). In particular, the relationship between species diversity and ecosystem services has a pivotal role in ecosystem functioning. Despite the increasing number of studies on the subject (e.g. [10–12]), we are far from unravelling the underlying processes.

Seed dispersal sustains a key ecosystem service by enabling movement of otherwise sessile plant individuals [13]. For fleshy-fruited tree species, seed dispersal is largely realized by avian frugivores [14–17], and both the number of seeds dispersed and the place where they are deposited depend upon the species identity of the frugivorous birds dispersing them [15,18]. Different species of birds have distinct meal sizes, gut retention times and foraging patterns [15,17,19,20], leading to complex seed dispersal distance patterns (*sensu* [21]). Some studies have explored how different frugivores contribute to the overall seed dispersal patterns and, in particular, their contribution to long-distance dispersal [18,22,23]. Indeed, the seeds dispersed over long distances have an increased probability of successful establishment compared with those remaining in the vicinity of the mother plants [24,25].

However, there are three persistent problems in studies of seed dispersal. First, setting the threshold between short- and long-distance dispersal is not straightforward and is mostly context-dependent. Second, some frugivorous species may have redundant roles and species diversity might not necessarily translate into functional diversity. Despite the substantial bibliography on the subject ([10,17,18] and references therein), further research is needed to enhance our understanding of the relationship between species identity, functional diversity and ecosystem functioning. Third, dispersal cannot be reduced to a unidimensional distance; rather, it is a complex process across heterogeneous landscapes. Here, we advocate that the combination of molecular markers and network techniques can contribute to further quantifying the role of functional diversity in shaping the fine spatial structure of seed dispersal.

## 2. Quantifying dispersal distances

In the last few years, ecologists have used molecular markers to unambiguously determine dispersal distances. This has resulted in a more detailed description of the dispersal kernel. In particular, the reported dispersal kernels are characterized by long tails [18]. This is reminiscent of power law distributions in complex systems where there is no dominant scale. This can be seen explicitly by considering a power law of the form:
2.1
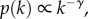
where *p*(*k*) is the probability of a seed reaching a distance *k* in arbitrary units, and *γ* is a critical exponent. The above relationship is called ‘scale free’ because the relationship between *k* and *p*(*k*) is not defined on a particular scale [26]. For example, if we represent the previous relationship on a log–log plot, the relationship appears as a straight line. It is invariant to a change in coordinates. The same relationship appears for small scales and for large scales. This does not happen for other types of relationships, such as an exponential one.

Examples of scale-free distributions include the frequency distribution of earthquakes releasing a particular energy. This relationship, known as the Gutenberg–Richter law, has a clear implication: we do not need mechanisms to explain small earthquakes different from those used to explain large ones. In seed dispersal studies, authors have used the distinction between short- and long-distance dispersal with different meanings and spatial scales in mind. Part of the confusion stems from the artificial distinction between these two scales. A dispersal kernel with long tails is indicative of the absence of a characteristic scale.

The above refers to the proper characterization of dispersal kernels. And yet this is only a simplified description of the services provided by frugivorous animals, as the process of seed dispersal takes place on both dimensions of the plane. In short, it is also important to understand the spatial pattern of seed dispersal. For example, is seed dispersal homogeneous in space? Does it tend to follow a major direction? Does it create seed aggregates? And if so, what are the determinants of such aggregates? To address these questions, we need a quantitative approach to map the spatial details of seed rain. Finally, if we want to unravel the differential contribution of different species in an attempt to estimate the functional diversity of seed dispersal, we need to identify the species responsible for each dispersal event.

## 3. A case study: a network approach to seed dispersal

Here we combine field sampling and observations, molecular analyses and analytical tools from network theory to advance our understanding of the spatial dynamics of seed dispersal. We focus on *Taxus baccata*, a temperate forest tree, and its avian seed dispersers' guild in a highly fragmented landscape in southern Spain. Specifically, we analyse a set of bird-dispersed seeds using highly polymorphic DNA markers (microsatellites) to identify their source tree. This information is then used to build two spatial networks of seed dispersal events (links) between source trees and seed-plots (nodes). The first network is constructed using local seed dispersal events, while the second also includes regional seed dispersal events. Once the seed dispersal networks are built, we characterize their structure applying network modularity analysis. Such an analysis finds, for each network, the best partition in modules, where a module is a subset of nodes from the network (here, mother trees and seed-plots) that interact much more frequently among themselves than they do with nodes from other modules [27,28]. The partition of each network in modules is based exclusively on the distribution of links between mother trees and seed-plots. Therefore, the activity of birds dispersing the seeds determines the modular structure of seed dispersal in the landscape. This approach allows a meaningful description of the spatial pattern of seed dispersal that can be related to the body of work bridging the structure of networks and their dynamics [29–32]. We proceed by using a series of ecological correlates to explore what variables better explain the observed modular structure. Finally, by comparing how the module assignment of nodes varies from the local to the regional dispersal network, we explore to what extent the incorporation of long-distance dispersal events (*sensu* [22]) transforms the local spatial pattern of seed dispersal, and to what degree this change is mediated by the behaviour of a particular functional group of frugivores.

## 4. Material and methods

### (a) Species and study site

The evergreen, non-resinous gymnosperm *T. baccata* L., the common yew, is a dioecious wind-pollinated tree growing across Europe [33]. Embryos are protected by pseudobayes composed of a seed partially covered by a red and fleshy aril (‘fruit’ hereafter, for simplicity). Fruits ripen asynchronously from late summer (August) to late autumn (November), but can remain on trees until late winter when not consumed by seed dispersers [19]. Yew relies essentially on avian frugivores for seed dispersal, especially on thrushes, *Turdus* spp., that feed on fruits directly from branches [19,34].

**Figure 1.**
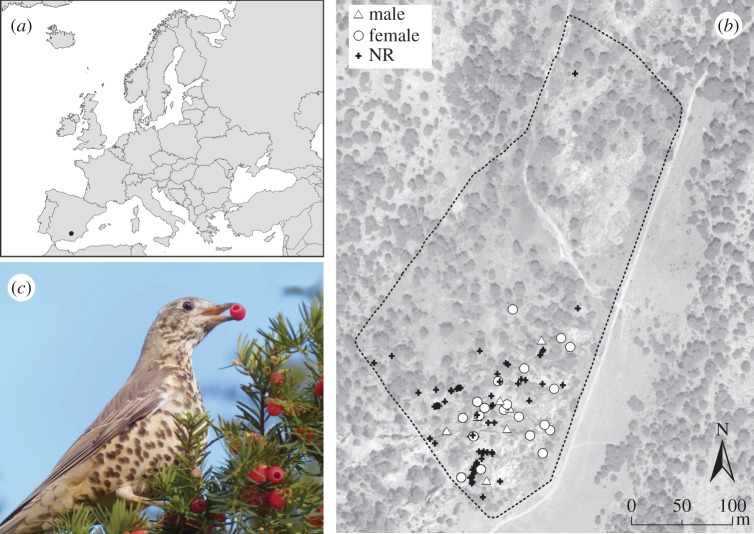
Geographical details of the study site. (*a*) Location of the study site in the Iberian Peninsula (black dot). (*b*) Map of the study site indicating the location of the *Taxus baccata* trees sampled within the fenced area (dotted line). White circle: *Taxus baccata* female trees; white triangle: *Taxus baccata* male trees; black cross: non-reproductive *T. baccata* individuals. (*c*) Mistle thrush (*Turdus viscivorus* handling a yew fruit, picture by Ralph Hancock).

The study population is located in Nava de las Correhuelas (37°55′ N, 2°51′ W, Parque Natural de las Sierras de Cazorla, Segura y las Villas) in the autonomous community of Andalusia, south-east Spain, at 1615 m a.s.l. elevation (figure 1; electronic supplementary material, figures S1 and S3). The site vegetation is dominated by grassland with scattered woody deciduous patches, with gravelly soil or rock outcrops covered by shrubs (e.g. *Juniperus* spp., *Rosa* spp.) or small isolated trees. Some pine stands (*Pinus nigra* subsp. *salzmannii* (Dunal) Franco) also occur on rocky slopes. The site is protected by a fence to exclude large mammals, and thus the grazing pressure is low. At this site, the yew grows as a secondary species and is found almost exclusively on rocks, aggregated in clusters. The highly fragmented landscape offers a rich mosaic of habitats at the local scale therefore providing an adequate system to study seed dispersal in heterogeneous conditions.

The field study was conducted from August to December 2006.

### (b) Frugivorous census

During 5 years, 2001–2002 and 2005–2007, we conducted direct observations of birds feeding on fruits. Eight focal trees were observed from hides under nearby trees, 40–50 m away, and with the help of 8 × 40 binoculars (Nikon, Monarch). Two-hour observation sessions were performed during the activity range of frugivores (09.00 to 18.00 h), such that each tree was observed at different times throughout the day. During observation, we focused on individual birds post-feeding behaviour and recorded the following data whenever possible: (i) bird species; (ii) flight distance to first perch; and (iii) identity of first perch after leaving the focal tree (for distance validation purposes; see electronic supplementary material, SI-2). We only considered here observations from legitimate seed dispersers (*sensu* [35]), excluding seed—or pulp—predators (e.g. *Parus* spp.).

### (c) Tree and seed sampling

In 2006, we censused 102 trees within the fenced study plot and identified 20 female trees, 6 male trees and 76 trees that were either juveniles or seedlings, and were classified as non-reproductive (figure 1*b*). Furthermore, we explored the surrounding landscape outside the fence, and censused 14 additional trees (electronic supplementary material, figure S1 and table S1). From those 14, five were male, six were female and three were non-reproductive. Leaf tissue was collected from each tree and remained in silica gel for a few days until the samples could be stored at −80°C. We preferentially collected newly formed leaves to increase the quality of the DNA when processing the samples for genetic analysis.

To represent the fine-scale environmental variability of the microsites where yew seeds are deposited, we defined eight categories of microhabitats based on soil type and vegetation cover: (1) TF, *T. baccata* female; (2) TM, *T. baccata* male; (3) S, shrub; (4) F, fleshy-fruited tree; (5) N, non-fleshy-fruited trees; (6) P, pine; (7) G, open ground; and (8) R, rock. We categorized types 1–6 as ‘covered’ microhabitats and types 7–8 as ‘open’ microhabitats (see electronic supplementary material, SI-1 for a detailed description of the microhabitats).

We studied the bird-generated seed rain with a microhabitat-based sampling of seed deposition. We collected the seeds deposited in seed traps that were 32 × 26 × 8 cm aluminium trays, top-covered with a wire mesh to prevent seed consumption by post-dispersal seed predators (mostly rodents, see [15] for a similar methodology). For the ‘rock’ microhabitat, due to the difficulty of installing seed traps and their conspicuousness to animals, we collected the seeds directly from open quadrats, permanently marked on the rock substrate, with the same surface as the seed traps. Open quadrats are prone to seed predation, however, it is usually low in open microhabitats and mostly occurs in late winter [36,37]. Nonetheless, we have accounted for this bias by counting some of the predated seeds found *in situ* as part of the seed rain. Although seed losses might have happened in the rock microhabitat, they were considered negligible (see also [34,38,39], for a validation of the methodology).

The sampling scheme consisted of an even number of sampling stations per microhabitat (when not constrained by microhabitat availability), with a total number of 277 (electronic supplementary material, figure S2). Each station consisted of two sampling surfaces—either seed traps or open quadrats—located at a maximum distance of 0.5 m of each other. Hereafter, for simplicity, we will refer to each sampling station as a seed-plot. We checked seed-plots monthly, collecting and counting all yew seeds during the whole fruiting season. Yew bird-dispersed seeds—smooth and without aril—are easily distinguishable from non-dispersed seeds in fallen fruits—seeds with aril remains. We estimated the bird-generated seed rain from the total number of seeds collected in seed-plots over all surveys. The collected seeds were then stored in silica gel.

To study the seed dispersal pattern, we processed all the seeds collected per microhabitat, with the exception of *T. baccata* female. Indeed, that was the only microhabitat in which we collected more than 100 seeds, and we randomly sub-sampled 143 seeds (electronic supplementary material, table S3). By doing so, we ensured that at least one seed per seed-plot was analysed to account for the spatial variability of the seed rain. For the 273 seeds selected, we carefully separated the endocarp tissue, of maternal inheritance, from the embryo and stored both at −80°C until processed for genetic analysis.

Each seed-plot, hence each seed, and *T. baccata* tree was georeferenced using a Leica GS20 differential GPS. Post processing using Leica GisDataPro software allowed for an average precision of 0.5 m. Original data in geographical coordinates were projected in UTM coordinates using ArcGis v. 10.2 software (ESRI, Redlands, CA, USA).

## 5. Genetic analyses

### (a) DNA extraction protocol and microsatellite genotyping

Briefly, frozen tissues—either leaf or endocarp—were ground using a zirconia-ball mill (Retsch Mixer Mill 200). The DNA was isolated using the DNeasy Plant Mini Kit (Qiagen Inc., USA) and 5 µl extract was used as a template for the polymerase chain reaction. Amplified fragments were then separated by capillary electrophoresis on an ABI 3730 sequencer, using the GS500LIZ size standard (Applied Biosystems). Results were recovered electronically, and all scorings were carried out using Genemapper v. 3.7 (Applied Biosystems) (from [40]). For full details on genotyping protocols see [41]. We performed a quality control screening protocol of our multilocus dataset following Selkoe & Toonen [42] recommendations.

### (b) Genetic diversity

Levels of microsatellite diversity (number of alleles per locus, *A*, and Nei's unbiased expected heterozygosity, *H*_E_; [43]), for both locally dispersed and immigrant seeds, were computed with GENETIX v. 4.05 [44].

### (c) Seed dispersal distance

To identify the source tree of each dispersed seed, we obtained the genotype at eight microsatellite loci (Simple Sequence Repeat (SSR) developed by Dubreuil *et al.* [45]) for all adult trees sampled, along with the multilocus genotype of the endocarp of the dispersed seeds. As some amplifications failed for several markers, we discarded the individuals that had more than one unamplified loci. Finally, our dataset included 254 endocarps (electronic supplementary material, table S3) and 26 female trees with at least seven amplified loci.

As the endocarp is maternally inherited, its genotype matches that of its mother tree [41]. Therefore, we searched the matching genotypes between each endocarp and the 26 candidate female trees for a complete set of at least seven microsatellite markers out of eight. Additionally, as the multilocus genotypes of the candidate female trees differed for at least one locus, we assumed that two seeds come from the same mother tree when the seeds' endocarp multilocus genotype are identical. We used GIMLET software [46] to identify each different multilocus genotype among the endocarps and to find the female tree multilocus genotype with which each of them match. When the endocarp did not match with any female tree, the seed was considered as immigrant.

For each dispersed seed, we calculated its dispersal distance. When the source tree was identified, we calculated the dispersal distance as the euclidean distance between the seed-plot containing that seed and its source tree. Similarly for the immigrant seeds, we calculated the minimum distance to the edge of the complementary area explored (electronic supplementary material, figure S1) using ArcGis v. 10.2 software (ESRI, Redlands, CA, USA).

## 6. Networks of seed dispersal

### (a) Building the networks

From the seed-female tree assignment, we built two seed dispersal networks in which female trees and seed-plot are represented as nodes linked by dispersal events—i.e. when a seed from a female tree is found in a seed-plot. The first network includes the locally dispersed seeds—i.e. from source trees located inside the fenced area—and will be, hereafter, referred to as the local network. The second network was constructed considering all the seed dispersal events, thus incorporating the seeds from mother trees located outside the fence and the immigrant seeds (i.e. from unidentified mother trees). This network will be, hereafter, referred to as the regional network.

### (b) Network analyses

Several algorithms to detect modules in networks are available (see [28,47–50]). Recently, the equation introduced by Barber [48] to calculate the modularity for bipartite networks was expanded by Dormann & Strauss [51], allowing both the matrix of observed links in the network and the matrix of expected links to be weighted (following [52]). In contrast, with the goal of maximizing modularity in qualitative networks (by maximizing the number of links within modules and minimizing the number of links among modules), modules are formed in quantitative networks by attempting to maximize the density of link weights within modules, and minimize the density of link weights among modules. Therefore, in the latter, modules are likely to form around the strongest interactions between nodes. Dormann & Strauss [51] have proposed an algorithm written in C^++^ and available through the open-source R-package ‘*bipartite*’ based on simulated annealing (QuanBiMo) to maximize weighted modularity in bipartite networks, which we used to perform the modularity analysis. The algorithm returns a global modularity value, *Q*, and the composition of the identified modules.

The modularity value, *Q*, cannot be used *per se* to compare different networks because the expected density of links within modules depends on network size (number of trees and seed-plots) as well as the number of links between trees and seed-plots, and the total number of seeds found in the seed-plots. Therefore, we used a null model to compare the observed value of modularity with the null model expectations. We used the null model implemented in the QuanBiMo algorithm to generate 1000 random networks with the same number of seeds contributed by each tree and the same number of seeds found in each seed-plot (i.e. the same marginal totals). We then computed the *p-*value for the local and regional networks as the fraction of those 1000 random networks having a modularity value equal or larger than the observed one.

In order to determine to what extent two seed-plots (trees) that are within the same module in the local network are also within the same module in the regional network, we computed the mutual information between the two assignments based on the variation of information introduced by Karrer *et al.* [53]. The variation of information between the modular structure of two networks is the sum of the information needed to describe the modular structure of the former network given the latter, and the information needed to describe the modular structure of the latter considering the former. Specifically, the variation of information between the local and regional networks for the set of nodes common to both is defined by
6.1
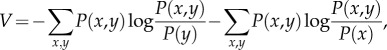
where *P*(*x*) is the fraction of nodes assigned to module *x* in the local network; *P*(*y*) is the fraction of nodes assigned to module *y* in the regional network; and *P*(*x, y*) is the fraction of nodes assigned to module *x* in the local network and to module *y* in the regional network. The variation index was computed separately for the set of trees (10), *V_m_*, and the set of seed-plots (49), *V_s_*, common to the local and regional network.

The above index goes from zero (the arrangement of the nodes within modules is the same across the two networks) to log *n* (each node constitutes its own module in one network, and all nodes are assigned to a single module in the other network), *n* being the number of nodes. As we are comparing networks of different sizes, we normalize this value by 1/log *n*, and therefore our measure of change in modular organization goes from zero to one.

### (c) Ecological correlates

We evaluate the role of microhabitat in shaping the modular organization of the networks using the *V* index (see above) for the set of seed-plots common to the two networks (i.e. 49). In this case, we assign the seed-plots to modules as a function of the microhabitat. That is, seed-plots belonging to the same microhabitat are assigned to the same module (figure 3*a*). Then we compute separately the variation index between the modular structure of the seed-plots based on microhabitat and seed-plots assignment to modules previously detected in the local network, *V*_hl_, and in the regional network, *V*_hr_. As before, the values are normalized and thus vary from zero to one. By comparing the variation in information between the microhabitat-based modularity and the modular organization of each network previously obtained by genetic analyses, we evaluate to what extent two seed-plots receiving seeds from the same mother tree, i.e. assigned to the same module, are also located in the same microhabitat.

Finally, we test the effect of geographical distance on the modular organization of the networks. For both networks independently, we compare the distribution of the between–seed-plot distances within modules with the distribution of all between seed-plot distances in the population by means of a Mann–Whitney *U* test. The analyses were performed using either the R package [54] or Matlab [55].

## 7. Results

### (a) Seed dispersal events

**Figure 2.**
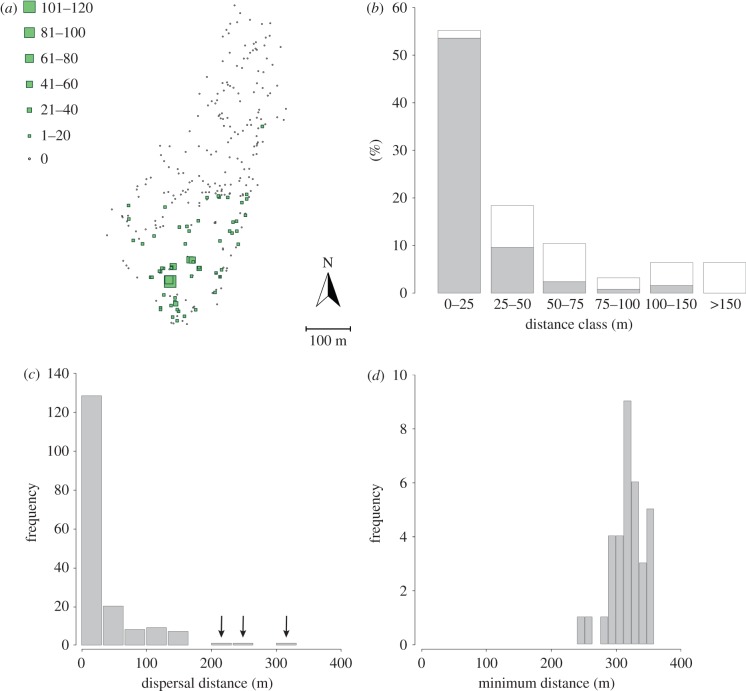
Empirical observations from our study system. (*a*) Seed rain of *T. baccata*. Squares represent seed-plots and are proportional to the number of yew seeds collected; dots represent seed-plots that did not receive *T. baccata* seeds. (*b*) Distribution of observed flight distances to first perch of frugivorous birds feeding on *T. baccata*. Grey bars represent short-distance seed dispersers, which include *Coccothraustes coccothraustes*, *Erithacus rubecula*, *Phoenicurus ochruros*, *Sylvia atricapilla* and *Turdus merula*. White bars represent long-distance seed dispersers, which include *Turdus viscivorus*, *T. philomelos* and *T. torquatus*. (*c*) Frequency distribution of the dispersal distances of seeds from known origin. All the seeds but three (indicated by an arrow) came from a source tree inside the fence. (*d*) Frequency distribution of the minimum dispersal distance of the seeds from unknown sources, i.e. the minimum distance between the seed-plot containing the immigrant seed and the limit of the complementary area explored (dotted line in the electronic supplementary material, figure S1).

Overall, the seed rain was highly heterogeneous, with 215 (77%) seed-plots receiving no *T. baccata* seeds; 47 (17%) receiving less than 10 seeds; and only 15 (5%) receiving 10 or more seeds (figure 2*a*). Additionally, all seeds were collected in the southern half of the fenced population, in the vicinity of yew trees (figures 1*b* and 2*a*), highlighting a spatially constrained seed dispersal pattern.

**Figure 3.**
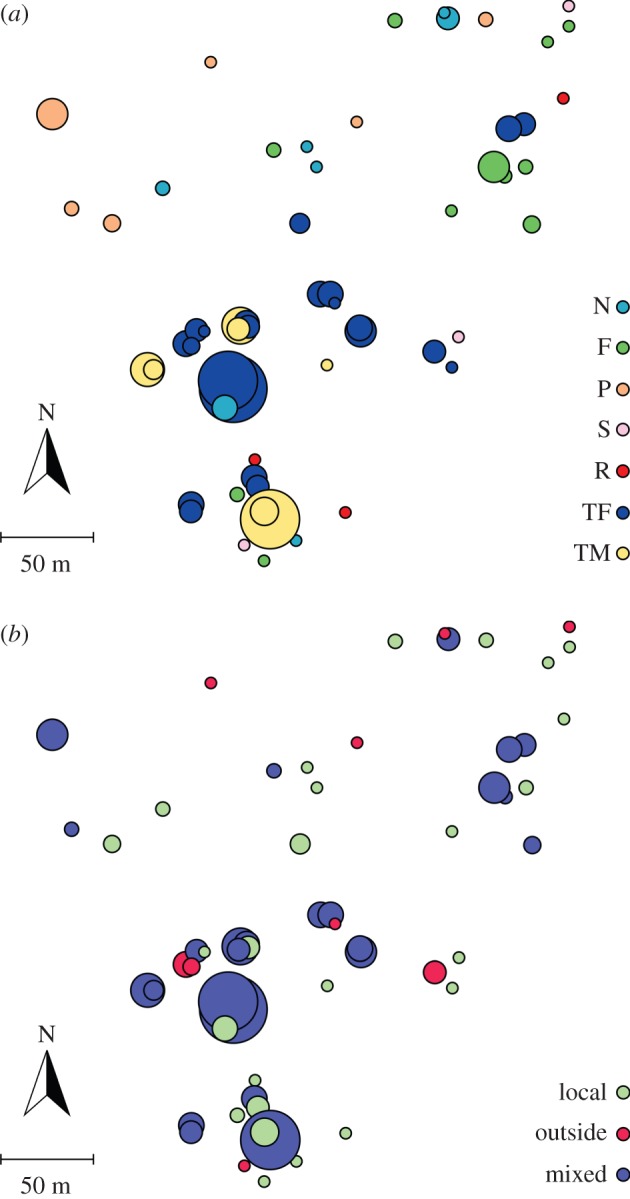
Spatial information of the seed-plots receiving bird-dispersed seeds. The size of the node is proportional to the total number of seeds analysed within each seed-plot (as in the regional network, figure 4*b*). (*a*) Microhabitat of the seed-plots. N, non-fleshy-fruited tree; F, fleshy-fruited tree; P, pine tree; S, shrub; R, rock; TF, *T. baccata* female tree; TM, *T. baccata* male tree (see the electronic supplementary material, SI-1 for a detailed description of the microhabitats). (*b*) Maternal origin of the seed pool analysed from each seed-plot. Local: seed-plots where all the seeds were from local mother trees; outside: seed-plots where all the seeds were from mother trees outside the fenced area; mixed: seed-plots receiving seeds from mother trees both inside and outside the fenced area.

The 254 seed endocarps analysed were distributed in 59 seed-plots, thus encompassing the spatial variability of the seed rain. From those endocarps, 172 (67.7%) matched a mother tree inside the fenced area, 3 (1.2%) matched a mother tree outside the fence and 79 (31.1%) originated from unknown sources (electronic supplementary material, tables S2 and S4). For clarity, we will refer to the seeds that originate from a mother tree inside the fence as the local seed pool, and from a mother tree outside the fence as the immigrant seed pool (figure 3*b*). Ten (42%) female trees contributed to the local seed pool, out of the 20 censused inside the fenced area. As for the immigrant seed pool, 1 of the 6 additional female trees sampled contributed 3 (4%) seeds, and 51 unidentified source trees—thus located outside our extended sampling area (electronic supplementary material, figures S1 and S3)—contributed the remaining 79 (96%) seeds.

Seed dispersal distances of seeds from a mother tree within the fenced area and from outside of it have different frequency distributions (figure 2*c,d*). Dispersal events inside the fence largely occur at very short distances (less than 50 m), in the vicinity of the source trees. Contrastingly, the seeds originating from outside the fence, and from unknown origin, displayed much longer dispersal distances (most of them greater than 300 m), especially because these distances are most probably underestimated (closest population located at 1.5 km; electronic supplementary material, figure S3).

There was an equal number of seed-plots, 25 (42%), receiving either strictly locally dispersed seeds or both locally dispersed and immigrant seeds (figure 3*b*). Only 9 (15%) seed-plots received immigrant seeds exclusively. No clear spatial pattern of the seed-plots depending on the origin of the seeds emerged, but we observed a tendency for the seed-plots with mixed seed origin to receive a greater number of seeds.

The immigrant seed pool displayed a higher mean allelic richness than the local seed pool (table 1), which probably reflects the larger number of mother trees (52 versus 10). Nonetheless, the lower observed heterozygosity, *H*_o_, among the immigrant seed pool suggests that they originate from genetically depauperate sources.

**Table 1.** Summary genetic data for the dispersed seeds. *n*, sample size; *N*_m_, number of identified mother trees; *A*, mean allelic richness; *H*_E_, Nei's unbiased expected heterozygosity; *H*_O_, observed heterozygosity. Standard deviations are reported in brackets.

seed-pool^a^	*n*	*N*_m_	*A*	*H*_E_	*H*_O_
all	254	62	9.0	0.713 (0.131)	0.522 (0.217)
local	172	10	6.375	0.687 (0.161)	0.542 (0.250)
immigrant	82	52	8.375	0.720 (0.087)	0.478 (0.165)

^a^Groups of seeds that originate either from a source tree inside, *local*, or outside, *immigrant*, the fenced area.

### (b) Frugivorous birds

During the 5 years of bird censuses, we identified eight bird species feeding on yew, four of them being thrushes (*Turdus* spp.). The frequency distributions of flight distances of individual bird species suggested two main foraging behaviours (electronic supplementary material, figure S4). On the one hand, we observed frequency distributions skewed towards short distances (less than 50 m) for *Coccothraustes coccothraustes*, *Erithacus rubecula*, *Phoenicurus ochruros*, *Sylvia atricapilla* and *Turdus merula*. On the other hand, *T. viscivorus*, *T. philomelos* and *T. torquatus* displayed kernels with longer tails. As these similarities are in agreement with the results of previous studies on similar assemblages of species [17,18,23], we regrouped the data in two categories: small-sized birds and medium-sized birds. A Kolmogorov–Smirnov test confirmed that the flight distance kernels of these two groups were indeed significantly different (*D* = 0.8426, *p* < 0.001).

In 2006, the year of the seed sampling, *C. coccothraustes*, *T. torquatus* and *T. philomelos* were not observed at the study site. Nonetheless, we observed these species only three, one and nine times, respectively, over the 5 years (from 125 records in total), and their absence might not affect the overall shape of the flight distance kernels of the two groups.

Frugivorous birds visited some of the female trees more often (electronic supplementary material, table S4), which might indicate a selective behaviour. Also, the most visited trees appear to contribute the highest number of seeds to the seed-plots. The results point towards an active selection of not only the microhabitat type but also individual source trees.

### (c) Seed dispersal network

**Figure 4.**
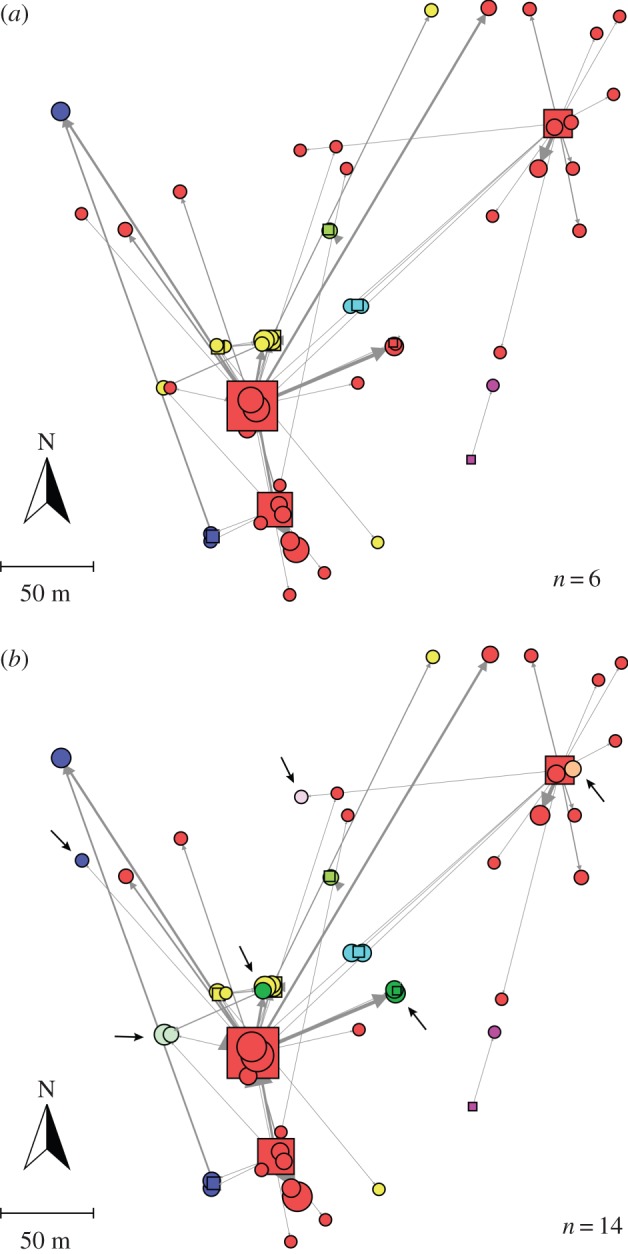
Modular organization of the seed dispersal network of *Taxus baccata*. Nodes represent mother trees (squares) and seed-plots (circles). The size of the node is proportional to the number of seeds either received, for seed-plots, or contributed, for mother trees. Arrows indicate seed dispersal events mediated by birds and are directed from the source, mother trees, to the destination, seed-plots. The same set of nodes is represented in both panels and include 10 mother trees and 49 seed-plots. Each colour represents a module, i.e. a group of non-overlapping highly connected mother trees and seed-plots detected by the QuanBiMo algorithm; *n* indicates the corresponding number of modules. (*a*) Modular organization of the local network (locally dispersed seeds). (*b*) Modular organization of the regional network (all seeds); black arrows indicate the nodes that have changed module assignment when incorporating regional dispersal events.

By comparing the modular structure of the set of nodes common to the two networks (10 trees and 49 seed-plots), we indirectly estimated how regional dispersal events (82 seeds) modify the spatial structure of the local seed rain. The analysis revealed a modular organization for the two networks (figure 4). However, the regional network was relatively more modular than the local network (*Q* = 0.583, *p* < 0.001 for the regional network, figure 4*b*; *Q* = 0.387, *p* = 0.015 for the local network, figure 4*a*) and had a much larger number of modules (14 for the regional network versus 6 for the local network).

So far, we have just compared the overall modularity of the local and regional networks. Two identical values of modularity, however, could be reached by different distributions of nodes into the different modules. Next, we quantified to what degree the ascription of nodes to modules changes once immigrant seeds are taken into account. Therefore, we explored whether two nodes belonging to the same module in the local network also belong to the same module in the regional network. The probability for two female trees within the same module in the local network to be assigned to the same module in the regional network was high (*V_m_* = 0.098). The equivalent probability of two seed-plots remaining in the same module after incorporating immigrant seeds was slightly lower (*V_s_* = 0.193), but still indicative of a similar organization of modules.

Once we compared the modular organization between the local and regional networks, we turned to the potential ecological correlates of this modular organization. Specifically, one could expect that nodes located within the same module belong to the same microhabitat type and/or are geographically closer. Regarding microhabitat, we did not find a tendency for the seed-plots receiving seeds from the same mother trees to be located in the same microhabitat, either for the local network (*V*_hl_ = 0.577) or the regional network (*V*_hr_ = 0.601). Regarding geographical distance, we compared the distribution of distances between seed-plots within a module with that among any two seed-plots within the entire network. Mann–Whitney *U* tests did not reveale significant differences in the local network (*W* = 321 610, *p* = 0.21), or the regional network (*W* = 335 270, *p* = 0.69). These results suggest that neither microhabitat type nor distance explain the observed modular organization of seed dispersal.

## 8. Discussion

By combining field work, molecular data and analytical tools from network theory, we have been able to get a glimpse of the differential contribution of short- and long-distance dispersers to the overall seed rain. Indeed, the results highlighted the coexistence of two complementary seed dispersal dynamics that might be driven locally by short-distance dispersers and regionally by long-distance dispersers.

Gene flow in plant population, through pollen or seed dispersal, is largely determined by landscape heterogeneity [30,34], therefore constraining the spatial structure of the seed rain. Our results are in agreement with the extensive literature on the subject (e.g. [18,23,34,39]), that is, high seed densities in microhabitats *Taxus* female, TF, and *Taxus* male, TM (figure 3*a*), and almost none in open microhabitats. However, here we were able to disentangle the origin of each seed and quantify the actual fraction of seeds resulting from long-distance dispersal (31.1%, *sensu* [22]), as well as the number of contributing mother trees (52; table 1). The results indicate an extensive seed flow between our studied population and other population patches within the landscape (electronic supplementary material, figure S3).

More importantly, our results enable the portrayal of the fine details of the spatial structuring of the seed rain beyond the average dispersal distance. Indeed, a network approximation to the spatial structure of seed dispersal has several advantages [56]. First, it facilitates the assessment of the simultaneous influence of all nodes beyond the information obtained from a series of pairwise comparisons between each tree—seed-plot pair. This is particularly relevant in other examples such as that of genetic variability, as one can prune the network by removing all links connecting nodes whose genetic similarity is mediated by their genetic similarity with other nodes [57–59]. Second, an advantage of looking at seed dispersal from a network perspective lies in the novel information that can be derived from the topology of such a network [56]. In particular, we can use quantitative tools such as modularity analysis. In our system, both the local and regional networks of seed dispersal events linking mother trees to seed-plots were organized in well-defined modules composed by a subset of seed-plots that received more seeds from the mother trees in the same module than from those outside this module. Indeed, the resulting modules can be seen as bottom-up classifications of meaningful evolutionary or conservation units [56,58,59]. This modular structure describes how variability is mapped in space, in contrast with dominant approaches looking at whether there is a significant variability [56].

The observed modular organization was caused neither by distance nor by the microhabitat type of the seed-plots. In fact, these two variables were partly accounted for when constructing the networks. As seed-plots receiving no seeds were not included in the networks (because they could not be linked to a mother tree), we excluded *a priori* those seed-plots avoided by frugivores. Indeed, these were mostly either located in microhabitats known to be avoided by birds (e.g. open ground, G), or at some distance from the fruiting trees [15,60]. The remaining subset of seed-plots included in the networks were thus inherently a combination of favoured microhabitat and distance [60].

Regarding the module composition itself, only the module assignment of the seed-plots slightly changed between the local and the regional networks (figure 4), most probably as a consequence of incorporating those seed-plots that received immigrant seeds exclusively (figure 3*b*). In the local and regional networks, the same two mother trees contributed the higher number of seeds to the seed-plots, and were the most connected (figure 4; electronic supplementary material, table S4). The origin of the seeds collected in the seed-plots around those two mother trees (figure 3*b*) and their high visitation rates (electronic supplementary material, table S4) suggest that they act as ‘frugivory hubs’ [61,62], and might therefore preferentially attract long-distance dispersers. Indeed, the higher relative modularity of the regional network supports the hypotheses that long-distance dispersers, here mostly mistle thrushes (*Turdus viscivorus*), are channeled through only a subset of the nodes (either trees or seed-plots). In autumn, during the fruiting season of yew at our study site, mistle thrushes' behaviour is principally driven by resource tracking and protection against predators [17,38,63]. This foraging behaviour would imply that the most productive trees and those with the biggest canopies would be preferentially visited, therefore, shaping the seed dispersal patterns at the landscape scale [62].

Thrushes are partial migrants [64], which makes them susceptible to the drastic environmental changes taking place on the Earth. Among the profound structural and functional modifications predicted [65], shifts in species distribution ranges due to climate change are expected to be a major driver of functional disruption [66]. Indeed, long-distance dispersal of *T. baccata* is largely dependent upon thrushes [23,34,38]. These species might modify their migration routes or become fully resident due to the expected global temperature rise [64]. Loosing this functional group could potentially be harmful for the yew by severely limiting its capacity to maintain a meta-population dynamic, which is indeed already declining [67].

## 9. Prospects for the future

The previous case study was missing a proper assignment of individual contributions for each seed dispersed. So far, genetic markers were used to determine distance from the mother tree. But we were lacking a specific assignment of frugivorous species to each event of seed dispersal. Thus, we relied on independent flight distance observations. This represents a first step, and in this paper, we argue that it may point towards the identification of two major functional groups. Nevertheless, we cannot unambiguously identify a frugivorous species behind each dispersal event. How to circumvent this? One formidable possibility is provided by the use of DNA barcoding [68]. This technique, introduced as a fast way to identify species by taking advantage of the diversity among DNA sequences, has most recently been used to unravel trophic interactions between species [69,70]. Samples of the dispersed seed could then not only identify the mother tree as here illustrated but also contain biological samples of the dispersal vector. This technique has already been proven to shed light on basic questions such as how species can coexist by partitioning their feeding niches [70]. More recently, DNA barcoding was developed to identify seed dispersers [71].

With an extension of DNA barcoding, therefore, one could unambiguously determine the bird species behind each dispersed seed. One could further characterize the functional diversity of a dispersal guild. The integration of molecular techniques to determine dispersal distances from mother trees, molecular techniques to identify the species dispersing this seed, and quantitative methods to describe the spatial mapping of seed dispersal can engender a true understanding of the spatial structuring of seed dispersal and how this is shaped by the functional diversity of frugivorous birds.

## Supplementary Material

Supporting Information

## Data accessibility

Data are available in the electronic supplementary material (tables S1 and S2).
