# Supplementary material for: How does the functional diversity of frugivorous birds shape the spatial pattern of seed dispersal? A case study in a relict plant species
Source: Philos Trans R Soc Lond B Biol Sci. 2016 May 19;371(1694):20150280. doi: 10.1098/rstb.2015.0280 (PMC4843700; doi:10.1098/rstb.2015.0280)
Supplement: Supporting Information [file rstb20150280supp1.pdf]

Supporting Information

Table S1: Summary data of *Taxus baccata* trees sampled at our study site.

Identity	Gender	Location	Coord X	Coord Y
C001	female	out	511632.63	4197793.62
C002	female	out	511763.1	4197869.26
C003	NR	out	511740.11	4197877.95
C004	female	in	511821.7	4197993.81
C005	female	in	511828.58	4198016.94
C006	female	in	511823.52	4198022.0
C007	female	in	511849.03	4198099.35
C008	female	in	511839.85	4198108.07
C009	male	in	511766.07	4197967.3
C010	female	in	511760.13	4197978.07
C011	NR	in	511749.9	4197969.98
C012	NR	in	511751.67	4197970.1
C013	NR	in	511753.25	4197974.19
C014	NR	in	511753.93	4197975.82
C015	NR	in	511752.52	4197977.78
C016	NR	in	511753.66	4197980.47
C017	female	in	511740.6	4197969.87
C018	NR	in	511761.27	4197988.6
C019	male	in	511726.32	4198016.25
C021	female	in	511742.22	4198028.93
C022	male	in	511757.11	4198030.39
C023	female	in	511758.71	4198031.18
C024	female	in	511752.92	4198010.7
C025	female	in	511786.07	4198042.22
C026	female	in	511777.3	4198065.52
C027	NR	in	511776.81	4198072.65
C028	NR	in	511800.76	4198067.29
C029	NR	in	511804.75	4198066.74
C030	female	in	511833.03	4198057.96
C031	NR	in	511821.67	4198062.87
C032	male	in	511786.24	4198018.67
C033	female	in	511797.99	4198030.07
C034	NR	in	511774.07	4198024.31
C035	female	in	511783.09	4198036.79
C036	NR	in	511779.21	4198045.95

Continued on next page

Table S1 – *Extended*

Identity	Gender [1]	Location [2]	Coord X [3]	Coord Y [3]
C037	male	in	511788.75	4198038.99
C038	NR	in	511779.55	4197966.32
C039	NR	in	511787.5	4198039.47
C040	NR	in	511808.76	4198046.26
C041	female	in	511763.85	4198038.86
C042	NR	in	511730.44	4198011.48
C043	NR	in	511853.96	4198370.89
C044	NR	in	511714.39	4198040.42
C045	NR	in	511759.92	4197977.88
C046	NR	in	511779.15	4198064.96
C047	male	in	511820.23	4198106.08
C048	NR	in	511654.56	4198083.72
C049	NR	in	511698.52	4198054.05
C050	NR	in	511716.02	4198056.98
C051	NR	in	511759.57	4198095.52
C052	NR	in	511787.49	4198082.25
C053	female	in	511803.16	4198078.07
C054	NR	in	511822.36	4198096.67
C055	NR	in	511821.53	4198095.24
C056	NR	in	511819.19	4198091.56
C057	NR	in	511714.79	4198003.75
C058	NR	in	511751.13	4198012.52
C059	NR	in	511709.89	4198008.59
C060	NR	in	511767.78	4198024.28
C061	female	in	511756.88	4198044.86
C062	NR	in	511797.41	4198063.24
C063	NR	in	511709.83	4198008.24
C064	NR	in	511730.72	4198056.46
C065	NR	in	511731.22	4198055.75
C066	NR	in	511736.41	4198057.94
C067	NR	in	511737.89	4198059.04
C068	NR	in	511738.42	4198058.7
C069	NR	in	511739.12	4198058.85
C070	NR	in	511739.62	4198058.71
C071	NR	in	511740.17	4198058.91
C072	NR	in	511772.6	4198041.7
C073	NR	in	511770.99	4198051.16
C074	NR	in	511771.01	4198040.71

Continued on next page

Table S1 – *Extended*

Identity	Gender [1]	Location [2]	Coord X [3]	Coord Y [3]
C075	NR	in	511771.76	4197995.11
C076	NR	in	511756.11	4197984.72
C077	NR	in	511756.79	4197984.66
C078	NR	in	511756.84	4197983.54
C079	NR	in	511751.93	4197977.51
C080	NR	in	511751.45	4197964.27
C081	NR	in	511751.98	4197965.57
C082	NR	in	511856.39	4198137.8
C083	NR	in	511671.94	4198090.79
C084	NR	in	511715.62	4198040.88
C085	NR	in	511716.84	4198041.38
C086	NR	in	511718.83	4198041.3
C087	NR	in	511716.6	4198040.93
C088	NR	in	511724.54	4198045.17
C089	NR	in	511755.88	4198010.78
C090	NR	in	511752.54	4198011.26
C091	NR	in	511752.56	4198011.83
C092	NR	in	511771.95	4197993.61
C093	NR	in	511766.53	4197995.15
C094	NR	in	511761.25	4197994.32
C095	NR	in	511763.15	4197995.97
C096	NR	in	511763.05	4197994.26
C097	NR	in	511758.69	4197995.96
C098	NR	in	511709.7	4198008.89
C099	female	in	511791.73	4198136.85
C100	NR	in	511756.9	4198031.9
C101	NR	in	511762.45	4197950.71
C102	NR	in	511749.92	4198010.63
C103	NR	in	511841.53	4198061.61
C104	NR	in	511785.16	4198083.46
C105	female	in	511808.96	4198011.49
C106	NR	in	511766.98	4197994.92
C107	NR	out	511791.07	4197751.24
C108	NR	out	511757.61	4197870.63
C109	male	out	512081.4	4198087.63
C110	male	out	512052.2	4198122.81
C111	female	out	512083.4	4198139.96
C112	male	out	512086.83	4198186.25

Continued on next page

Table S1 – *Extended*

Identity	Gender [1]	Location [2]	Coord X [3]	Coord Y [3]
C113	male	out	512082.15	4198204.05
C114	female	out	512059.7	4198183.11
C115	female	out	512533.12	4198693.67
C116	female	out	512542.1	4198701.85
C117	male	out	512510.04	4198650.62

[1] NR: not reproductive.

[2] Either inside the fenced area, *in*, or outside of it, *out*; see Fig. 1B for a map of these areas.

[3] Geographic coordinates, in UTM.

Table S2: Maternal composition of the seed rain analysed within each seed-plot.

Seed-plot	Microhabitat [1]	Seeds [2]	Self (n) [3]	Local (n) [3]	Outside (n) [3]	Coord X [4]	Coord Y [4]
F01	F	0	-	-	-	511844.49	4198154.24
F02	F	0	-	-	-	511830.8	4198144.47
F04	F	0	-	-	-	511812.33	4198142.84
F05	F	0	-	-	-	511821.18	4198155.95
F06	F	0	-	-	-	511808.91	4198132.05
F08	F	2	-	C023 (2)	-	511809.24	4198134.89
F10	F	0	-	-	-	511780.94	4198146.89
F11	F	0	-	-	-	511759.3	4198137.96
F12	F	2	-	C007 (1)	new1 (1)	511768.03	4198090.86
F14	F	0	-	-	-	511732.56	4198093.68
F16	F	0	-	-	-	511703.43	4198084.6
F20	F	1	-	C007 (1)	-	511861.17	4198127.4
F22	F	1	-	C007 (1)	-	511868.35	4198132.76
F25	F	2	-	C007 (2)	-	511853.66	4198084.94
F26	F	2	-	-	C111 (1), new12 (1)	511846.53	4198082.03
F27	F	8	-	C007 (6)	new1 (1), new12 (1)	511842.89	4198085.04
F30	F	3	-	C007 (2)	new11 (1)	511855.76	4198065.42
F31	F	0	-	-	-	511844.83	4198048.64
F32	F	0	-	-	-	511838.13	4198025.94
F42	F	0	-	-	-	511756.13	4197948.37
F48	F	2	-	C010 (2)	-	511755.64	4197974.04
F49	F	0	-	-	-	511777.6	4197982.08
F50	F	1	-	C007 (1)	-	511828.45	4198070.34
F51	F	0	-	-	-	511759.28	4197947.34
F52	F	1	-	C024 (1)	-	511764.75	4197951.43
F56	F	0	-	-	-	511821.65	4198275.03
F57	F	0	-	-	-	511824.92	4198289.53
F60	F	0	-	-	-	511926.74	4198267.05
F61	F	0	-	-	-	511913.75	4198254.38
F62	F	0	-	-	-	511923.46	4198250.52
F65	F	0	-	-	-	511918.79	4198359.11
F68	F	0	-	-	-	511777.41	4198202.88
F70	F	0	-	-	-	511704.62	4198146.87

Continued on next page

Table S2 – *Extended*

Seed-plot	Microhabitat [1]	Seeds [2]	Self (n) [3]	Local (n) [3]	Outside (n) [3]	Coord X [4]	Coord Y [4]
F72	F	0	-	-	-	511795.8	4198256.36
F73	F	0	-	-	-	511860.89	4198187.62
F74	F	0	-	-	-	511872.2	4198177.69
F77	F	0	-	-	-	511866	4198361.35
F78	F	0	-	-	-	511891.7	4198240.77
F79	F	0	-	-	-	511821.75	4198098.7
G02	G	0	-	-	-	511867.6234	4198153.3053
G03	G	0	-	-	-	511874.3629	4198144.7273
G04	G	0	-	-	-	511871.8329	4198165.3374
G05	G	0	-	-	-	511864.7541	4198176.9794
G06	G	0	-	-	-	511797.1219	4198211.3036
G08	G	0	-	-	-	511809.0285	4198205.1538
G09	G	0	-	-	-	511822.801	4198203.4157
G10	G	0	-	-	-	511842.8619	4198194.8201
G12	G	0	-	-	-	511860.8522	4198090.5603
G14	G	0	-	-	-	511838.9367	4198029.5359
G16	G	0	-	-	-	511810.8903	4198154.5258
G18	G	0	-	-	-	511797.6082	4198167.0778
G20	G	0	-	-	-	511787.0103	4198159.6835
G23	G	0	-	-	-	511717.7305	4198087.5641
G24	G	0	-	-	-	511732.1238	4198100.162
G25	G	0	-	-	-	511783.2226	4198157.5158
G26	G	0	-	-	-	511773.2314	4198145.0105
G32	G	0	-	-	-	511745.198	4198142.062
G34	G	0	-	-	-	511717.7251	4198154.6186
G36	G	0	-	-	-	511705.6568	4198151.762
G41	G	0	-	-	-	511833.0034	4198251.8234
G45	G	0	-	-	-	511869.0884	4198243.0735
G47	G	0	-	-	-	511840.5803	4198217.2323
G50	G	0	-	-	-	511845.1121	4198223.2209
G51	G	0	-	-	-	511829.5902	4198252.6999
G54	G	0	-	-	-	511840.2192	4198302.954
G56	G	0	-	-	-	511861.4615	4198327.8417
G58	G	0	-	-	-	511831.4377	4198260.275

Continued on next page

Table S2 – *Extended*

Seed-plot	Microhabitat [1]	Seeds [2]	Self (n) [3]	Local (n) [3]	Outside (n) [3]	Coord X [4]	Coord Y [4]
G60	G	0	-	-	-	511848.6401	4198298.5162
G61	G	0	-	-	-	511850.6102	4198232.3674
G62	G	0	-	-	-	511855.3178	4198229.2724
G64	G	0	-	-	-	511862.6124	4198234.423
G69	G	0	-	-	-	511874.9074	4198243.0983
G70	G	0	-	-	-	511873.3453	4198250.5636
G71	G	0	-	-	-	511883.4243	4198253.7893
G72	G	0	-	-	-	511885.7857	4198261.6813
G73	G	0	-	-	-	511887.0084	4198265.6951
G74	G	0	-	-	-	511899.7135	4198286.7406
G76	G	0	-	-	-	511903.2872	4198290.231
G78	G	0	-	-	-	511913.7251	4198301.5838
N01	N	0	-	-	-	511830.79	4198138.89
N02	N	5	-	C024 (4)	new12 (1)	511827.27	4198135.5
N03	N	0	-	-	-	511797.94	4198126.89
N05	N	0	-	-	-	511777.39	4198142.79
N11	N	0	-	-	-	511718.9	4198162.39
N13	N	0	-	-	-	511707.37	4198136.08
N15	N	0	-	-	-	511691.68	4198107.91
N16	N	0	-	-	-	511692.31	4198092.98
N17	N	2	-	C024 (2)	-	511730.31	4198077.7
N22	N	1	-	C024 (1)	-	511779.34	4198091.92
N28	N	0	-	-	-	511852.34	4198060.72
N29	N	0	-	-	-	511830.34	4197999.69
N32	N	0	-	-	-	511772.74	4197951.1
N33	N	1	-	C010 (1)	-	511775.64	4197958.06
N34	N	0	-	-	-	511779.35	4197946.35
N36	N	0	-	-	-	511764.92	4197958.26
N37	N	0	-	-	-	511764.87	4197990.49
N38	N	0	-	-	-	511744.85	4197989.9
N39	N	0	-	-	-	511733.5	4197981.16
N40	N	6	-	C023 (1), C024 (5)	-	511751.43	4198003.23
N41	N	0	-	-	-	511815.21	4198198.9
N42	N	0	-	-	-	511836.33	4198172.44

Continued on next page

Table S2 – *Extended*

Seed-plot	Microhabitat [1]	Seeds [2]	Self (n) [3]	Local (n) [3]	Outside (n) [3]	Coord X [4]	Coord Y [4]
N44	N	0	-	-	-	511857.38	4198159.77
N45	N	0	-	-	-	511858.79	4198163.9
N46	N	0	-	-	-	511835.45	4198139.15
N47	N	1	-	-	new12 (1)	511826.02	4198137.31
N48	N	0	-	-	-	511797.87	4198122.43
N51	N	1	-	C010 (1)	-	511782.62	4198085.06
N56	N	0	-	-	-	511690.06	4198099.33
N57	N	0	-	-	-	511660.3	4198078.76
N60	N	0	-	-	-	511667.48	4198117.19
N63	N	0	-	-	-	511783.42	4198233.75
N65	N	0	-	-	-	511753.48	4198211.25
N66	N	0	-	-	-	511740.64	4198198.15
N68	N	0	-	-	-	511739.3	4198212.01
N70	N	0	-	-	-	511745.3	4198189.13
N73	N	0	-	-	-	511725.35	4198164.7
N76	N	0	-	-	-	511692.37	4198123.08
N79	N	0	-	-	-	511682.72	4198107.48
N80	N	0	-	-	-	511683.51	4198105.4
P01	P	0	-	-	-	511854.72	4198192.97
P04	P	0	-	-	-	511843.61	4198240.69
P05	P	0	-	-	-	511834.39	4198241.62
P06	P	0	-	-	-	511839.68	4198253.55
P07	P	0	-	-	-	511826.5	4198256.23
P08	P	0	-	-	-	511810.58	4198272.08
P10	P	0	-	-	-	511798.07	4198289.54
P11	P	0	-	-	-	511834.21	4198294.3
P12	P	0	-	-	-	511809.03	4198314.59
P14	P	0	-	-	-	511853.79	4198330.94
P15	P	0	-	-	-	511864.11	4198341.89
P18	P	0	-	-	-	511854.15	4198395.91
P20	P	0	-	-	-	511858.65	4198409.46
P21	P	0	-	-	-	511903.41	4198394.4
P22	P	0	-	-	-	511902.5	4198375.91
P24	P	0	-	-	-	511896.74	4198373.18

Continued on next page

Table S2 – *Extended*

Seed-plot	Microhabitat [1]	Seeds [2]	Self (n) [3]	Local (n) [3]	Outside (n) [3]	Coord X [4]	Coord Y [4]
P26	P	0	-	-	-	511894.87	4198361.42
P28	P	0	-	-	-	511898.31	4198342.01
P29	P	0	-	-	-	511906.72	4198332.62
P32	P	0	-	-	-	511893.33	4198321.52
P33	P	0	-	-	-	511885.66	4198323.14
P35	P	0	-	-	-	511883.66	4198305.12
P36	P	0	-	-	-	511879.09	4198291.78
P40	P	0	-	-	-	511892.15	4198298.11
P42	P	1	-	-	new12 (1)	511796.29	4198100.31
P44	P	0	-	-	-	511785.95	4198093.96
P47	P	0	-	-	-	511757.19	4198127.38
P48	P	1	-	-	new13 (1)	511746.64	4198120.73
P52	P	3	-	C024 (3)	-	511713.16	4198065.98
P54	P	2	-	C024 (4)	new14 (1)	511699.38	4198071.12
P56	P	8	-	C017 (3), C024 (4)	new14 (1)	511692.83	4198102.88
P62	P	0	-	-	-	511802.17	4198142.39
P64	P	0	-	-	-	511773.91	4198171.38
P68	P	0	-	-	-	511766.97	4198170.69
P69	P	0	-	-	-	511752.32	4198180.11
P71	P	0	-	-	-	511843.25	4198141.31
P76	P	2	-	C007 (2)	-	511840.09	4198135.09
P77	P	0	-	-	-	511836.05	4198153.98
P78	P	0	-	-	-	511828.19	4198152.51
P80	P	0	-	-	-	511803.21	4198159.52
R02	R	0	-	-	-	511862.68	4198138.18
R03	R	0	-	-	-	511862.42	4198139.88
R04	R	0	-	-	-	511859.07	4198137.36
R08	R	0	-	-	-	511858.7	4198111.54
R09	R	0	-	-	-	511866.33	4198110.99
R10	R	1	-	C007 (1)	-	511866.48	4198108.43
R14	R	0	-	-	-	511821.68	4198096.86
R15	R	0	-	-	-	511820.33	4198097.17
R17	R	0	-	-	-	511788.5	4197971
R18	R	0	-	-	-	511790.3	4197972.45

Continued on next page

Table S2 – *Extended*

Seed-plot	Microhabitat [1]	Seeds [2]	Self (*n*) [3]	Local (*n*) [3]	Outside (*n*) [3]	Coord X [4]	Coord Y [4]
R19	R	1	-	C021 (1)	-	511792.39	4197967.78
R20	R	0	-	-	-	511786.3	4197965.26
R21	R	1	-	C010 (1)	-	511761.63	4197985.57
R23	R	0	-	-	-	511754.21	4197988.13
R29	R	0	-	-	-	511753.12	4198027.97
R31	R	0	-	-	-	511684.47	4198040.69
R33	R	0	-	-	-	511689.23	4198033.69
R34	R	0	-	-	-	511692.33	4198029.51
R38	R	0	-	-	-	511705.31	4198015.91
R42	R	0	-	-	-	511831.18	4198147.05
R44	R	0	-	-	-	511824.59	4198144.79
R45	R	0	-	-	-	511821.95	4198141.21
R47	R	0	-	-	-	511878.22	4198198.52
R50	R	0	-	-	-	511883.09	4198199.65
R54	R	0	-	-	-	511879.57	4198211.01
R56	R	0	-	-	-	511883.31	4198212.31
R58	R	0	-	-	-	511884.01	4198215.05
R60	R	0	-	-	-	511885	4198217.22
R63	R	0	-	-	-	511892.26	4198217.97
R65	R	0	-	-	-	511883.11	4198226.41
R67	R	0	-	-	-	511885.64	4198238.05
R68	R	0	-	-	-	511896.14	4198243.96
R70	R	0	-	-	-	511889.82	4198251.61
R71	R	0	-	-	-	511869.59	4198342.05
R72	R	0	-	-	-	511873.47	4198350.58
R73	R	0	-	-	-	511877.62	4198354.1
R74	R	0	-	-	-	511881.56	4198356.75
R75	R	0	-	-	-	511883.43	4198354.96
R76	R	0	-	-	-	511878.82	4198363.93
R77	R	0	-	-	-	511882.09	4198366.23
S02	S	1	-	-	new12 (1)	511868.33	4198139.65
S04	S	0	-	-	-	511842.78	4198140.36
S07	S	0	-	-	-	511765.57	4198177.2
S08	S	0	-	-	-	511770.18	4198139.39

Continued on next page

Table S2 – *Extended*

Seed-plot	Microhabitat [1]	Seeds [2]	Self (*n*) [3]	Local (*n*) [3]	Outside (*n*) [3]	Coord X [4]	Coord Y [4]
S10	S	0	-	-	-	511743.2	4198105.89
S11	S	0	-	-	-	511781.42	4198127.3
S12	S	0	-	-	-	511761.87	4198132.94
S14	S	0	-	-	-	511739.56	4198090.94
S15	S	0	-	-	-	511733.69	4198089.41
S16	S	0	-	-	-	511824.21	4197975.86
S17	S	1	-	C007 (1)	-	511830.86	4198027.28
S19	S	0	-	-	-	511871.93	4198135.77
S20	S	0	-	-	-	511884.53	4198153.57
S22	S	0	-	-	-	511813.28	4198151.1
S24	S	0	-	-	-	511807.39	4198142.77
S25	S	0	-	-	-	511751.62	4198143.23
S28	S	0	-	-	-	511743.97	4198092.42
S29	S	0	-	-	-	511708.03	4198073.57
S37	S	0	-	-	-	511833.67	4198030.62
S40	S	0	-	-	-	511877.39	4198162.51
S42	S	0	-	-	-	511828.01	4198258.46
S44	S	0	-	-	-	511816.16	4198278.36
S46	S	0	-	-	-	511824.18	4198279.76
S47	S	0	-	-	-	511832.3	4198305.48
S48	S	0	-	-	-	511839	4198306.49
S50	S	0	-	-	-	511832.54	4198337.89
S52	S	0	-	-	-	511843.04	4198350.62
S54	S	0	-	-	-	511832.61	4198374.72
S55	S	0	-	-	-	511841.5	4198379.48
S56	S	0	-	-	-	511838.13	4198384.75
S57	S	0	-	-	-	511843.86	4198393.75
S60	S	0	-	-	-	511850.24	4198421.06
S65	S	0	-	-	-	511873.24	4198399.57
S69	S	0	-	-	-	511891.06	4198393.32
S71	S	0	-	-	-	511843.59	4198301.07
S72	S	0	-	-	-	511856	4198289.96
S73	S	0	-	-	-	511858.53	4198278.26
S75	S	0	-	-	-	511822.94	4197997.71

Continued on next page

Table S2 – *Extended*

Seed-plot	Microhabitat [1]	Seeds [2]	Self (n) [3]	Local (n) [3]	Outside (n) [3]	Coord X [4]	Coord Y [4]
S76	S	0	-	-	-	511817.38	4197992.77
S80	S	1	-	-	new15 (1)	511758.04	4197956.83
TF01	TF	0	-	-	-	511821.43	4197994.1
TF02	TF	0	-	-	-	511821.13	4197993.64
TF03	TF	1	-	C004 (1)	-	511828.58	4198016.94
TF04	TF	0	-	-	-	511828.58	4198016.94
TF05	TF	5	-	-	new16 (5)	511822.63	4198022.56
TF06	TF	0	-	-	-	511823.04	4198021.48
TF07	TF	5	C007 (3)	-	C111 (1), new21 (1)	511853.15	4198099.66
TF08	TF	6	C007 (5)	-	new21 (1)	511847.95	4198098.23
TF11	TF	6	C010 (4)	C024 (1)	new22 (1)	511761.4	4197979.73
TF12	TF	5	C010 (4)	C024 (1)	-	511762.59	4197976.68
TF13	TF	6	C017 (2)	C010 (1)	new23 (1), new24 (2)	511740.02	4197970.6
TF14	TF	5	C017 (1)	C010 (1)	new25 (1), new26 (1),<br>new27 (1)	511739.95	4197968.18
TF17	TF	3	-	-	new28 (1), new29 (1),<br>new31 (1)	511740.08	4198024.29
TF18	TF	6	-	-	new30 (1), new32 (1),<br>new33 (2), new34 (1)<br>new36 (1)	511738.23	4198025.21
TF19	TF	1	C021 (1)	-	-	511744.47	4198029.4
TF20	TF	5	C021 (1)	C024 (1)	new35 (1), new37 (1),<br>new38 (1)	511741.86	4198029.71
TF23	TF	5	C023 (4)	C021 (1)	-	511759.36	4198030.81
TF24	TF	6	C023 (3)	C024 (1)	C111 (1), new39 (1)	511758.79	4198032.07
TF25	TF	21	C024 (10)	C007 (1), C010 (4)	new6 (1), new9 (1),<br>new10 (1), new11 (1)<br>new12 (1), new47 (1)	511754.32	4198009.88
TF26	TF	18	C024 (11)	C007 (1), C021 (1),<br>C023 (1)	new17 (2), new18 (1)<br>new19 (1)	511752.51<br>511752.51	4198012.35<br>4198012.35
TF27	TF	6	C025 (2)	C007 (1)	new13 (1), new40 (1),<br>new41 (1)	511783.86	4198042.09
TF28	TF	6	C025 (3)	-	new40 (1), new42 (1), (1)<br>new43 (1)	511787.28<br>511787.28	4198041.8<br>4198041.8

Continued on next page

Table S2 – *Extended*

Seed-plot	Microhabitat [1]	Seeds [2]	Self (*n*) [3]	Local (*n*) [3]	Outside (*n*) [3]	Coord X [4]	Coord Y [4]
TF29	TF	0	-	-	-	511777.56	4198065.23
TF30	TF	4	C026 (4)	-	-	511776.92	4198065.96
TF33	TF	0	-	-	-	511833.02	4198057.53
TF34	TF	0	-	-	-	511832.83	4198059.14
TF37	TF	8	C033 (1)	C024 (6)	new7 (1)	511797.62	4198029.29
TF38	TF	6	-	C024 (1)	new6 (1), new7 (1), new44 (1), new45 (1) new46 (1)	511797.28	4198030.51
TM09	TM	18	-	C010 (14)	new48 (1), new49 (1), new50 (1), new51 (1)	511766.83	4197965.46
TM10	TM	7	-	C010 (6), C024 (1)	-	511764.89	4197968.06
TM15	TM	9	-	C010 (1), C023 (2)	new2 (1), new3 (1), new5 (1), new30 (2), new33 (1)	511725.18	4198016.18
TM16	TM	4	-	C024 (1)	new2 (1), new4 (2)	511727.23	4198016.17
TM21	TM	10	-	C021 (1), C023 (4), C024 (4)	new8 (1)	511756.7	4198031.23
TM22	TM	5	-	C021 (1), C023 (1), C024 (1)	new6 (1), new7 (1)	511756.09	4198030.01
TM31	TM	0	-	-	-	511780.12	4198046.7
TM32	TM	1	-	-	new20 (1)	511788.75	4198038.99
TM35	TM	0	-	-	-	511787.84	4198018.71
TM36	TM	1	-	C024 (1) -		511786.17	4198017.77

[1] TF: *Taxus baccata* female tree; TM: *Taxus bacata* male tree; F: fleshy-fruited tree; N: non fleshy-fruited tree; G: open ground; R: rock; S: shrub.

[2] Total number of seeds analysed within individual seed-plots

[3] Identity of the mother trees that contributed seeds to the seed-plots. Self: seeds from the mother tree under which the seed-plot is located; local: seeds from a mother tree within the population; outside: seeds from a mother tree located outside the fence; number of seeds contributed by each mother tree in brackets. Mother trees identified as *new* are unidentified source trees. See the "Material and methods" section for further details.

[4] Geographic coordinates, in UTM, of the seed-plots, therefore of the seeds.

Table S3: Seed sampling scheme by microhabitat for genetic analyses

Microhabitat†	TF	TM	F	N	P	R	S	G
N seeds collected [1]	514	58	29	19	19	3	3	0
N seeds genotyped [2]	143	57	29	19	19	3	3	0
N endocarps [3]	134	55	25	17	17	3	3	0

† See Supplementary Material 1 for a detailed description of the microhabitats

[1] Total number of seeds collected in seed-plots

[2] Number of seeds selected for microsatellite genotyping

[3] Final number of endocarps analysed after accounting for microsatellite amplification failure

Table S4: Summary data on birds' visitation rate and seed contribution to the seed-plots of *T. baccata* female trees at our study site. Female trees neither observed nor contributing seeds are not included.

Female tree	Visitation rate [1]	Contributed seeds
C004	not observed	1
C006	0.5	0
C007	5.83	29
C010	5.08	40
C017	0.5	6
C021	1.03	7
C023	0.46	18
C024	2.01	61
C025	1.52	5
C026	not observed	4
C033	not observed	1
C111	not observed	3

[1] Mean number of bird visits per hour

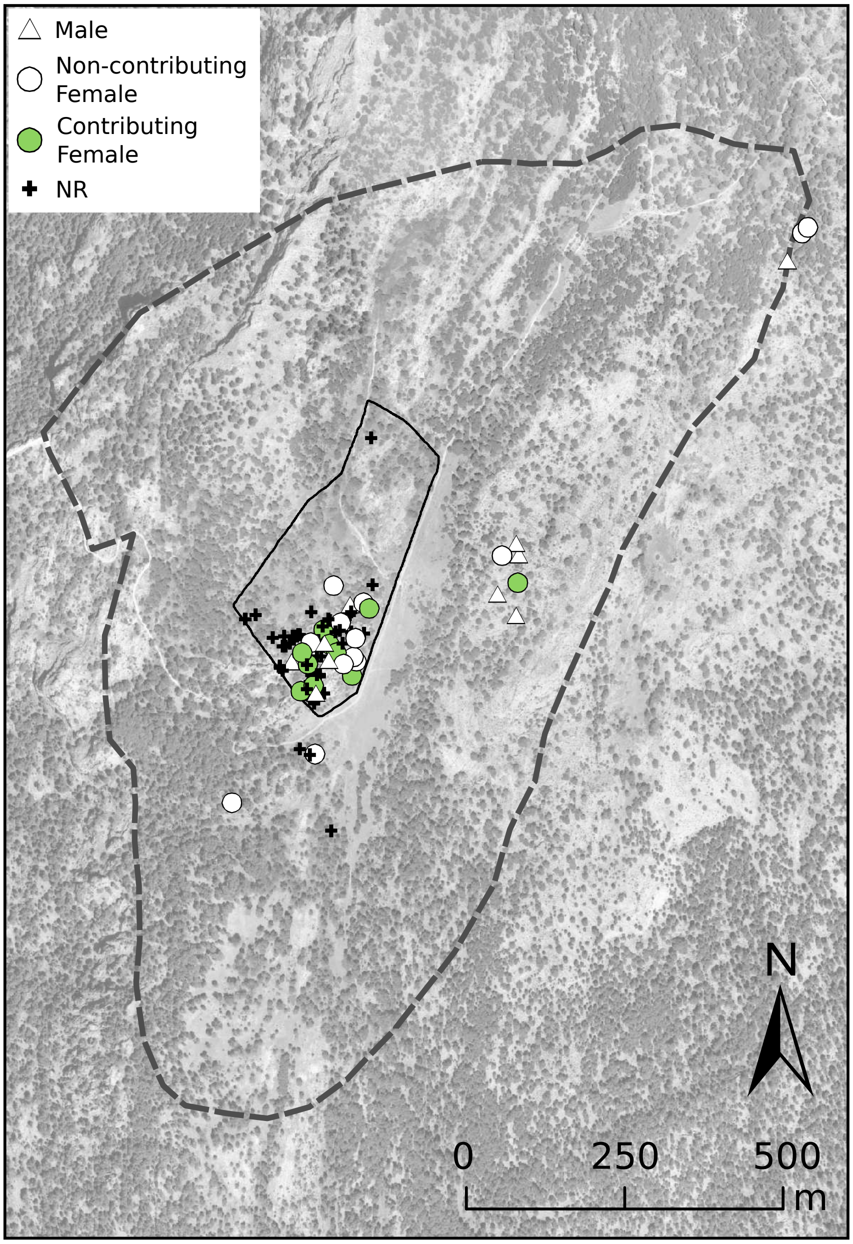

Figure S1: Map of the study site indicating the location of the *Taxus baccata* trees sampled within the fenced area (black line) and the additional area explored (dotted line). Green circle: *Taxus bacata* female trees contributing seeds in seed-plots; white circle: *Taxus bacata* female trees not contributing seeds in seed-plots; white triangle: *Taxus bacata* male trees; black cross: non-reproductive *Taxus baccata* individuals.

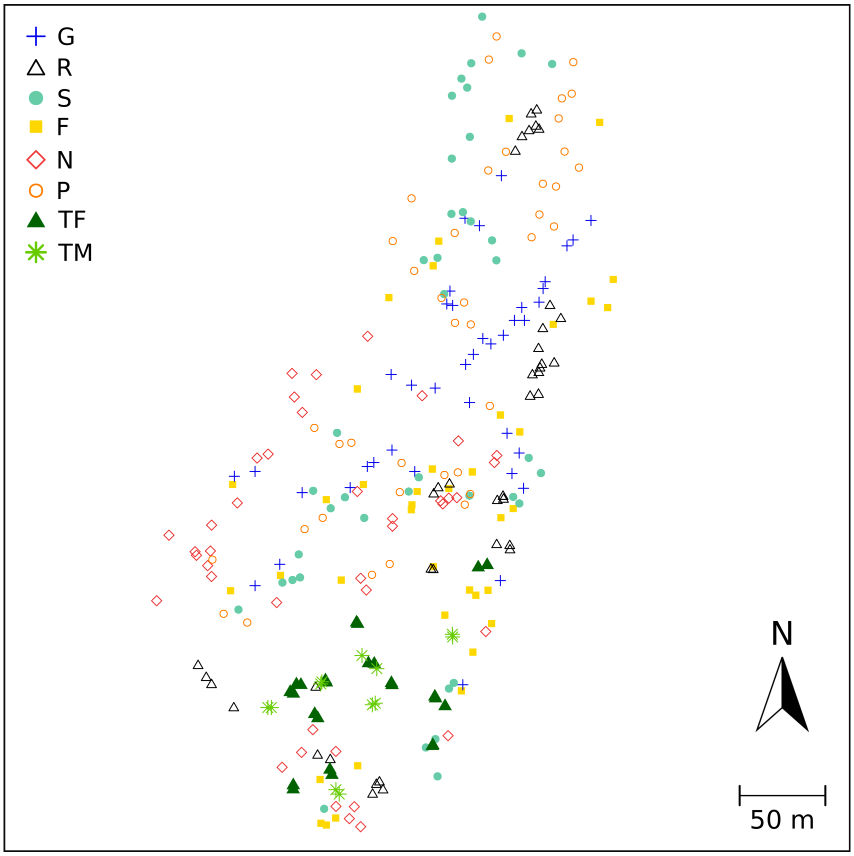

Figure S2: Spatial distribution of the seed-plots within the studied population (inside the fence as in Fig. S1) and their microhabitat. G: open ground; R: rock; S: shrub; F: fleshy-fruited tree; N: non fleshy-fruited tree; P: pine tree; TF: *Taxus baccata* female tree; TM: *Taxus bacata* male tree (see Supplementary Material 1 for a detailed description of the microhabitats).

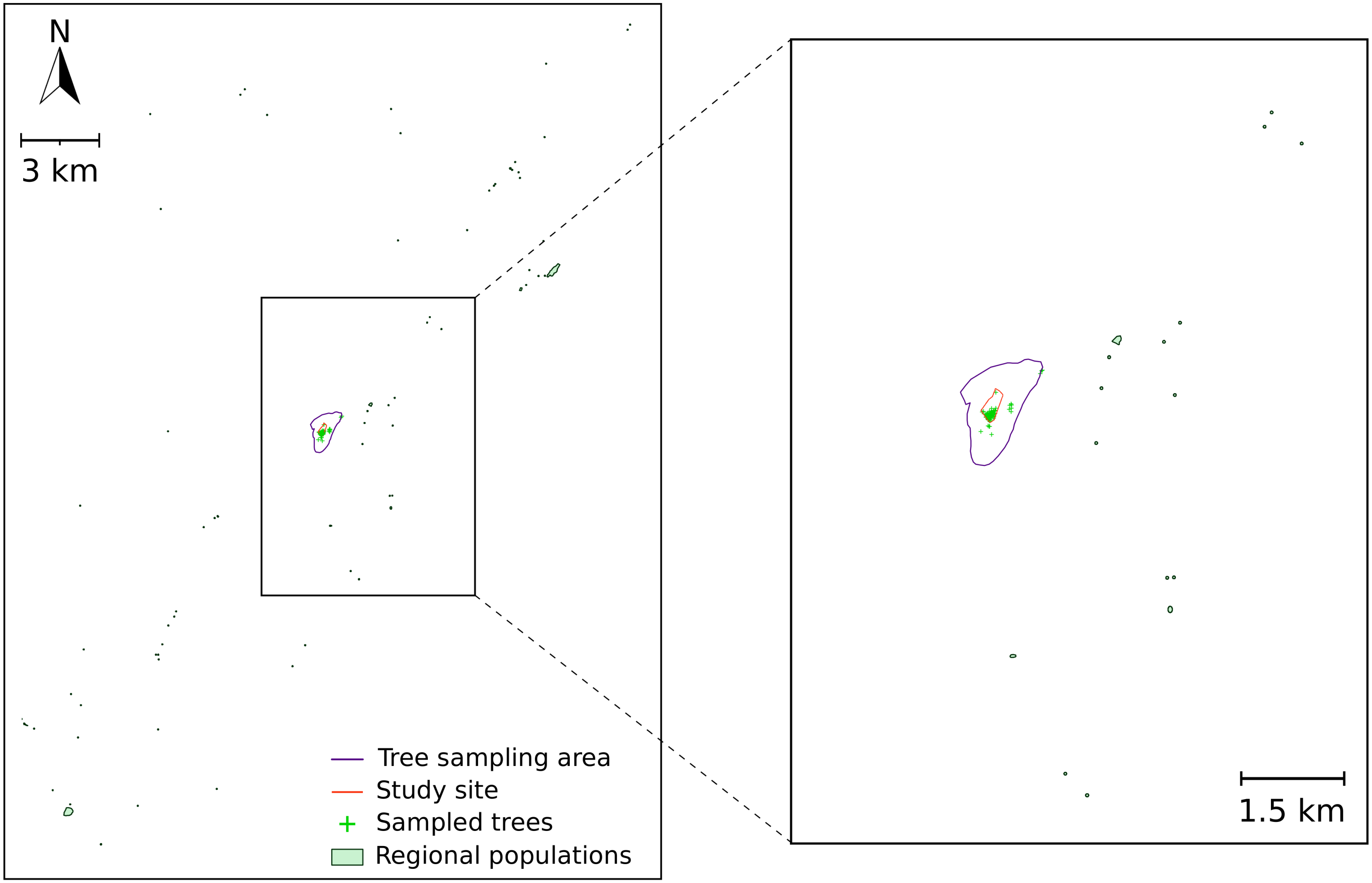

Figure S3: Regional map of *Taxus baccata*. Source: Parque Natural de Las Sierras de Cazorla, Segura y Las Villas.

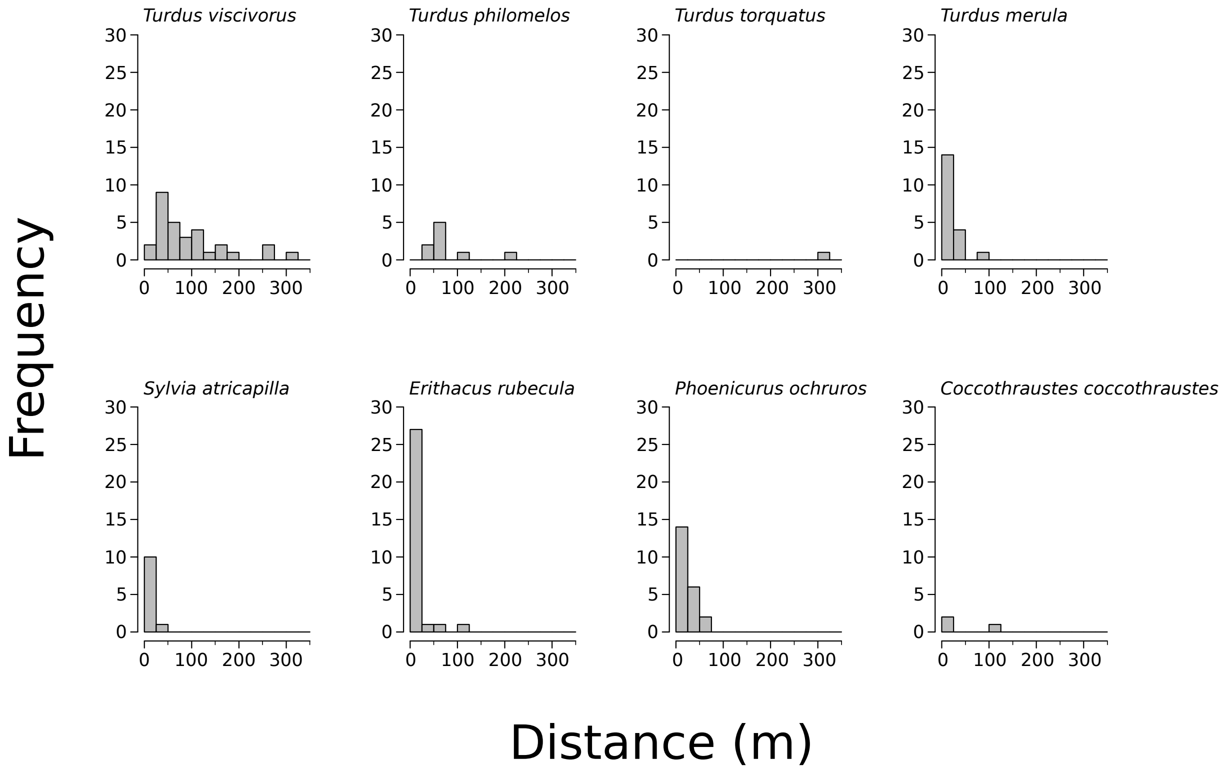

Figure S4: Frequency distribution of flight distances observations for individual bird species at the study site.

SI-1 Microhabitats

Given the type of soil and the vegetation cover, we defined the following microhabitat categories for seed sampling:

1) TF: "*Taxus baccata* female," beneath a female conspecific tree;
2) TM: "*Taxus baccata* male," beneath a male conspecific tree;
3) S: "shrub," beneath an individual of a woody species $<$1.50 m in height (e.g. *Rosa* spp.);
4) F: "fleshy-fruited tree," beneath a tree $>$1.50 m in height, other than the yew, that produce fleshy fruits (e.g. *Prunus mahaleb* L.);
5) N: "non-fleshy-fruited trees," beneath a tree $>$1.50 m of a non fleshy-fruited species (e.g. *Quercus* spp.);
6) P: "pine," beneath *Pinus nigra* subsp. *salzmannii*;
7) G: "open ground," deep soil with cover of non-woody vegetation (pasture) or barren soil with gravel under 25 cm^2;
8) R: "rock," including stones on soil, boulders, and rock substrates of at least 25 cm^2.

SI-2 Bird watching protocol

Bird watching was conducted from hides under nearby trees 40–50 m away and with the help of 8×40 binoculars (Nikon, Monarch). We followed each individual bird from the moment it entered a focal tree and until it stopped on a first perch or until out of view. We recorded the following data whenever possible: (1) bird species; (2) flight distance to first perch; and (3) identity of first perch after leaving the focal tree (for distance validation purposes). The flight distances to the first perch were estimated visually and validated a posteriori (see "Trees and seed sampling" section for more information).

Prior to bird observations, all seed-plots and *Taxus baccata* trees in the study population were mapped. When flights were recorded between any two georeferenced points, we compared afterwards the value estimated from direct observation with the actual Euclidean distance. Moreover, to avoid distance estimates bias towards short distances, we accounted for birds that flew out of view; these observations represented 6.4% (8 out of 125) of all the recorded distances. When in the field, we estimated that these birds had flown a minimum distance of 100 m. We thus picked a random distance between 100 m and 350 m (see Godoy & Jordano, 2001; Martínez *et al.*, 2008, for dispersal kernel estimates of a similar assemblage of species).

References

Godoy, J. A. & Jordano, P. (2001). Seed dispersal by animals: exact identification of source trees with endocarp DNA microsatellites. *Molecular Ecology*, **10**, 2275–2283.

Martínez, I., García, D. & Obeso, J. R. (2008). Differential seed dispersal patterns generated by a common assemblage of vertebrate frugivores in three fleshy-fruited trees. *Ecoscience*, **15**, 189–199.
